# CCAAT-enhancer binding protein-α (C/EBPα) and hepatocyte nuclear factor 4α (HNF4α) regulate expression of the human fructose-1,6-bisphosphatase 1 (FBP1) gene in human hepatocellular carcinoma HepG2 cells

**DOI:** 10.1371/journal.pone.0194252

**Published:** 2018-03-22

**Authors:** Siriluck Wattanavanitchakorn, Pinnara Rojvirat, Tanit Chavalit, Michael J. MacDonald, Sarawut Jitrapakdee

**Affiliations:** 1 Department of Biochemistry, Faculty of Science, Mahidol University, Bangkok, Thailand; 2 Division of Interdisciplinary, Mahidol University, Kanjanaburi, Thailand; 3 Childrens Diabetes Center, University of Wisconsin School of Medicine and Public Health, Madison, WI, United States of America; Rosalind Franklin University of Medicine and Science, UNITED STATES

## Abstract

Fructose-1,6-bisphosphatase (FBP1) plays an essential role in gluconeogenesis. Here we report that the human FBP1 gene is regulated by two liver-enriched transcription factors, CCAAT-enhancer binding protein-α (C/EBPα) and hepatocyte nuclear factor 4α (HNF4α) in human hepatoma HepG2 cells. C/EBPα regulates transcription of FBP1 gene via binding to the two overlapping C/EBPα sites located at nucleotide -228/-208 while HNF4α regulates FBP1 gene through binding to the classical H4-SBM site and direct repeat 3 (DR3) located at nucleotides -566/-554 and -212/-198, respectively. Mutations of these transcription factor binding sites result in marked decrease of C/EBPα- or HNF4α-mediated transcription activation of FBP1 promoter-luciferase reporter expression. Electrophoretic mobility shift assays of -228/-208 C/EBPα or -566/-554 and -212/-198 HNF4α sites with nuclear extract of HepG2 cells overexpressing C/EBPα or HNF4α confirms binding of these two transcription factors to these sites. Finally, we showed that siRNA-mediated suppression of C/EBPα or HNF4α expression in HepG2 cells lowers expression of FBP1 in parallel with down-regulation of expression of other gluconeogenic enzymes. Our results suggest that an overall gluconeogenic program is regulated by these two transcription factors, enabling transcription to occur in a liver-specific manner.

## Introduction

The liver plays an important role in maintaining glucose homeostasis [1, 2]. During feeding, an increase in blood glucose triggers pancreatic beta cells to release insulin which acts on the liver to stimulate glycogen synthesis while inhibiting gluconeogenesis. In contrast, during fasting, low plasma glucose stimulates pancreatic alpha cells to release glucagon which acts on the liver by suppressing glycolysis and stimulating glycogen breakdown and gluconeogenesis [3, 4]. The latter pathway constitutes > 90% of hepatic glucose production during prolonged fasting [5]. Fructose-1,6-bisphosphatase (FBP) is one of the four gluconeognic enzymes. FBP catalyzes the dephosphorylation of fructose-1,6-bisphosphate to fructose-6-phosphate. Fructose-6-phosphate is then converted to glucose-6-phosphate immediately before being terminally converted to glucose by glucose-6-phosphtase. FBP activity is allosterically inhibited by AMP and fructose-2,6-bisphosphate [6,7]. The latter allosteric molecule is produced by the bifunctional enzyme, 6-phosphofructo-2-kinase/fructose-2,6-bisphosphatase (6PFK2/FBP2) when the level of insulin is high [8]. FBP is comprised of two isoforms, FBP1 and FBP2 [9, 10]. FBP1 is expressed in gluconeogenic tissues while FBP2 is expressed in skeletal muscle where it supports glycogen synthesis [11, 12]

Several studies in rodent models show that increased FBP1 is associated with diabetes [13–17]. Similar findings were also reported for humans with type 2 diabetes [18]. In addition to diabetes, FBP1 was also reported to be a tumor suppressor gene. Loss of function expression of FBP1 in many cancers results in accumulation of fructose-1,6-bisphosphate, increasing the levels of glycolytic intermediates which in turn drives the Warburg effect [19–22]. In the past decade, FBP1 draws an attention of being an attractive anti-diabetic drug because several FBP1 inhibitors have been reported, many of which can reduce plasma glucose during fasting and postprandial periods in obese and non-obese type 2 diabetic animals [23–28]. Although accumulating data show that overexpression of FBP1 is associated with hyperglycemia in diabetic patients, little is known how FPB1 expression is regulated at transcriptional level. Although the human FBP1 gene promoter has been cloned and some *cis*-acting elements that mediate basal transcription of FBP1 have been reported [29–30], neither of the study identified the transcription factors that implicate in hepatocyte-specific or energy metabolism. Here we identified for the first time that the hepatocyte nuclear factor 4α (HNF4α) and CCAAT-enhancer binding protein-α (C/EBPα) are important transcriptional regulators for FBP1 expression in HepG2 cells.

## Materials and methods

### Generation of human FBP1 promoter-luciferase reporter constructs and site-directed mutagenesis

The 886 nucleotides upstream of transcription start site together with the first 20 nucleotides downstream of transcription site of human FBP1 gene (-886/+20) were cloned from genomic DNA by a PCR technique using the hFBP1 forward and reverse primers (sequences shown in Table 1) designed from human FBP1 gene sequence deposited at NCBI (accession no. NT_008470). Five 5’-truncated hFBP1 gene promoter fragments consisting of 520, 420, 320, 220 and 120 nucleotides were generated by PCR using forward oligonucleotide primers that direct to different nucleotide positions at the 5’-end of the FBP1 promoter and the common reverse primer (hFBP1-Rev) using full length (906 bp hBP1) as a template. Oligonucleotide primers used to generate these mutants are shown in Table 1. The PCR was carried out in a 50 μl reaction mixture containing 1x PCR reaction buffer (20 mM Tris-HCl pH 8.4, 50 mM KCl), 0.2 mM dNTP mixture, 1.5 mM MgCl_2_, 0.5 μM each primer, 50 ng genomic DNA and 2.5 units *Taq* DNA polymerase in an automated thermal cycler MJ Mini (Bio-Rad). The PCR profile consisted of initial denaturation at 94°C for 5 min followed by 30 cycles of denaturation at 94°C for 45 sec, annealing at 60°C for 45 sec and extension at 72°C for 2 min, and final extension at 72°C for 10 min. The PCR products were digested with *Sac*I and *Xho*I before ligation into *Sac*I and *Xho*I sites of the pGL4 luciferase reporter vector (Promega).

**Table 1:** Oligonucleotides used for generating wild type and mutant reporter constructs, EMSA and qPCR.

Oligonucleotide	Sequence (5’-3’)	Construct/Gene name
**5’-truncation constructs**		
hFBP1-F	GAGCTCAAGCTTTTACTGAGGCCTCTGC	pGL4-886 hFBP1
hFBP1-R	CTCGAGGCTCCGCCTGCTTGGATCT	
-500hFBP1-F	GAGCTCCTCACATCTTGGAAATTCAAATACT	pGL4-500 hFBP1
hFBP1-R	CTCGAGGCTCCGCCTGCTTGGATCT	
400hFBP1-F	GAGCTCAGAAA CGGGGACTCTGTGTC	pGL4-400 hFBP1
hFBP1-R	CTCGAGGCTCCGCCTGCTTGGATCT	
-300hFBP1-F	GAGCTCCACGGGGCAGGAGCTGCA	pGL4-300 hFBP1
hFBP1-R	CTCGAGGCTCCGCCTGCTTGGATCT	
-200hFBP1-F	GAGCTCACTTCCGTT TTATGATTTTGGAGG	pGL4-200 hFBP1
hFBP1-R	CTCGAGGCTCCGCCTGCTTGGATCT	
-100hFBP1-F	GAGCTCGGGTGTGTGTGGGGGGCG	pGL4-100 hFBP1
hFBP1-R	CTCGAGGCTCCGCCTGCTTGGATCT	
**Site-directed mutagenesis**		
MutC/EBPα1 (-233/-204)-F	GGTGGCACATATGTTGTTACTTAACCTTTC	ΔC/EBPα1–300 hFBP1
MutC/EBPα1(-233/-204)-R	GAAAGGTTAAGTAACAACATATGTGCCACC	
MutC/EBPα2 (-228/-199)-F	CATTGAGCAAGCATA TGTTCCTTTCTGAAC	ΔC/EBPα2–300 hFBP1
MutC/EBPα2 (-228/-199)-R	GTTCAGAAAGGAACATATGCTTGCTCAATG	
MutC/EBPα1 and 2 (-228/-199)-F	CACATATGTTGCATATGTTCCTTTCTGAAC	ΔC/EBP1αΔC/EBPα2–300 hFBP1
MutC/EBPα1 and 2 (-228/-199)-R	GTTCAGAAAGGAACATATGCAACATATGTG	
MutHNF4α1 (-577/-548)-F	GTGGAGCCCTCTCATATGTGTGTGGTAGCC	ΔHNF4α1–886 hFBP1
MutHNF4α1 (-577/-548)-R	GGCTACCACACACATATGAGAGGGCTCCAC	
MutHNF4α2 (-367/-337)-F	AGAAGGGCCAGGCATATGCTTAGGCAGAGTG	ΔHNF4α2–886 hFBP1
MutHNF4α2 (-367/-337)-R	CACTCTGCCTAAGCATATGCCTGGCCCTTCT	
MutHNF4α3 (-222/-189)-F	GCAAGTTACTTAACCATATGGAACTTCCGTTTTA	ΔHNF4α3–886 hFBP1
MutHNF4α3 (-222/-189)-R	TAAAACGGAAGTTCCATATGGTTAAGTAACTTGC	
**EMSA**		
C/EBPα (-228/-208)-F*	GGTGGCATTGAGCAAGTTACTTAACCTTTCT	Probe and wild type competitor
C/EBPα (-228/-208)-R	AGAAAGGTTAAGTAACTTGCTCAATGCCACC	
C/EBP consensus-F*	CTCGCCTATTGCGCAAGGGGCCGGATC	
C/EBP consensus-R	GATCCGGCCCCTTGCGCAATAGGCGAG	
HNF4α1 (-569/-549)-F*	CCTCTGGCCTTTGTGTGGTAG	
HNF4α1 (-569/-549)-R	CTACCACACAAAGGCCAGAGG	
HNF4α2 (-361/-341)-F*	GGCCAGGTGACAGGCCAGGCA	
HNF4α2 (-361/-341)-R	TGCCTGGCCTGTCACCTGGCC	
HNF4α3 (-216/-194)-F*	TACTTAACCTTTCTGAACTTCCG	
HNF4α3 (-216/-194)-R	CGGAAGTTCAGAAAGGTTAAGTA	
**qPCR**		
Exon3 hFBP1-F	AGCCTTCTGAGAAGGATGCTC	FBP1
Exon3 hFBP1-R	GTCCAGCATGAAGCAGTTGAC	
PC-For	GATGACTTCACAGCCCAG	PC
PC-Rev	GGGCACCTCTGTGTCCAG	
PEPCK-C-F	CCACAGCGGCTGCAGAACAT	PEPCK-C
PEPCK-C-R	GAAGGGCCGCATGGCAA	
G6PC-F	GGGAAAGATAAAGCCGACCTAC	G6Pase
G6PC-R	CAGCAAGGTAGATTCGTGACAG	
HNF4-F	CAGGCTCAAGAAATGCTTCC	HNF4α
HNF4-R	GGCTGCTGTCCTCATAGCTT	
C/EBPα -F	TGGACAAGAACAGCAACGAGTA	C/EBPα
C/EBPα-R	ATTGTCACTGGTCAGCTCCAG	

*3′ labeled with biotin

Underline; restriction enzyme site

The binding sites of C/EBPα (-218/-208 and -228/-218) or HNF4α (-212/-198, -359/-346 and -566/-554) in the hFBP1 promoter-luciferase reporter were mutated by Quick change site-directed mutagenesis (Stratagene Agilent Technologies) using the 320 nucleotides fragment of hFBP1 or the 906 bp nucleotides of hFBP1 promoter-reporter construct as template. The mutagenesis reaction was performed by PCR as described previously [31]. The primers used for site-directed mutagenesis are shown in Table 1. The clones containing corrected mutations were verified by nucleotide sequencing (Macrogen, South Korea).

### Construction and expression of plasmids overexpressing C/EBPα or HNF4α protein

The full coding sequence of C/EBPα cDNA was PCR-amplified from the rat C/EBPα cDNA clone [32] using C/EBPα forward (5’-AAGCTTATGGAGTCGGCCGACTTCTAC-3’) and reverse primers (5’-CTCGAGTCACGCGCAGTTGCCCATGGC-3’) using the PCR conditions as described above. The PCR products were then digested with *Hind*III and *Xho*I before ligation into *Hind*III and *Xho*I sites of the pcDNA3 expression vector (Invitrogen). A bacterial and mammalian expression plasmid for 6Histidine tagged human HNF4α was used to prepare human HNF4α as described previously [31].

### Cell culture, transient transfection and reporter assays

The human hepatocellular carcinoma cell line, HepG2 cells (ATCC: HB-8065) were obtained from Professor John Wallace, University of Adelaide, Australia. Cells were maintained in a complete medium [high glucose Dulbecco's modified Eagle's medium (DMEM) (Gibco)] supplemented with 28 mmol/l NaHCO_3_, 10% (v/v) heat-inactivated fetal bovine serum (Gibco), 100 units/ml penicillin-streptomycin (Gibco) at 37°C in 5% CO_2_ atmosphere. For transient transfection, 1x10^5^ cells were seeded into a 24-well cell culture plate containing 0.5 ml of antibiotic free-complete DMEM. After 24 h, cells were transfected with 0.2 pmol each of the hFBP1 promoter-luciferase reporter constructs and pRSV-β-gal vector expressing β-galactosidase and 0.1 pmol of plasmid encoding rat C/EBPα (pcDNA3-C/EBPα) or human HNF4α (pcDNA3-HNF4α) or empty vector (pcDNA3) using Lipofectamine^TM^ 2000 reagent (Invitrogen) as described previously [31]. The transfected cells were incubated at 37°C in 5% CO_2_ for 48 h prior to harvesting for subsequent analysis. Luciferase enzyme activity was measured using luciferase assay reagent (Promega) and β-galactosidase activity was assayed using ONPG as substrate as described previously [31].

For overexpression studies, 5x10^5^ HepG2 cells were seeded into a 6-well cell culture plate before transfection with 1.6 pmol of C/EBPα-pcDNA3, hHNF4α-pcDNA3 or empty vector in the presence of 10 μl of Lipofectamine 2000 reagent. The transfected cells were maintained as described above before being harvested for Western blot analysis and EMSA.

### Electrophoretic mobility-shift assays (EMSAs) and ChIP assays

Nuclear proteins were prepared from HepG2 cells overexpressing C/EBPα. Cells were washed once with cold PBS, scraped off and centrifuged at 3000 x g at 4°C for 5 min. The cell pellet was resuspended in 1 ml of cold buffer 1, containing 10 mM HEPES-KOH buffer, pH 7.9, 1.5 mM MgCl_2_, 10 mM KCl, 0.5 mM DTT, 1x protease inhibitor (Roche) and incubated on ice for 15 min. Fifty microlitres of 10% (v/v) NP-40 was added to the cell suspension before centrifugation at 3000x g at 4°C for 3 min. The pellet was resuspended in 50 μl of cold buffer 2 containing 20 mM HEPES-KOH buffer, pH 7.9, 25% (v/v) glycerol, 0.2 mM EDTA, 10 mM KCl, 1.5 mM MgCl_2_ and 420 mM NaCl, and incubated on ice for 40 min. After centrifugation at 12,000 x g at 4°C for 15 min, the supernatant was collected and stored at -80°C until used. The 3’-labeled biotinylated oligonucleotides (Biobasic, Canada) were annealed with their unlabeled complementary strands in 1x annealing buffer (10 mM Tris pH 7.4, 1 mM EDTA and 50 mM NaCl). The oligonucleotides used in EMSA are shown in Table 1. The DNA-protein binding reaction was performed in a 20 μl-reaction mixture containing 1x binding buffer (15mM HEPES pH 7.9, 50mM KCl, 1mM MgCl_2_), 2 μg of poly (dI-dC), 0.05% NP-40, 2.5% (v/v) glycerol, 60–480 fmole of biotinylated labeled double-stranded oligonucleotide probe and 5 μg of nuclear extract or 0.2 μg of purified 6xHis hHNF4α [31] at 4°C for 30 min. For the competition assays, excess amounts of unlabeled double stranded oligonucleotide (competitor) were included in the binding reaction mixture at 4°C for 30 min before adding probe. For supershift assays, 0.2 μg of rabbit anti-C/EBPα (sc-61) polyclonal antibody (SantaCruz Biotech) or anti-HNF4α (sc-8987) polyclonal antibody (Santa Cruz Biotech) was included in the reaction mixture at 4°C for 30 min before adding probe. The DNA-protein complexes were analyzed by 5% non-denaturing polyacrylamide gel electrophoresis followed by electroblotting. The DNA-protein complexes were then detected using Lightshift Chemiluminescent EMSA kit (Pierce) and visualized by Gel Doc System (GeneTools).

### siRNA transfections

5 x 10^6^ HepG2 cells were transfected with 5 nM siRNA target to human C/EBPα or human HNF4α (Qiagen) using X-treamGene siRNA transfection reagent (Roche). At 48 h post-transfection, the transfected cells were harvested and total RNAs were extracted. Expression of C/EBPα, HNF4α, pyruvate carboxylase (PC), PEPCK-C, G6Pase1 and FBP1 were analyzed by qPCR as described above. The PCR primers used for detection expression of these genes are shown in Table 1.

### Quantitative real time RT-PCR (qPCR)

Total RNA was isolated from HepG2 or Huh7 cells using TRIzol®Reagent (Gibco) according to manufacturer’s instructions. Reverse transcription was performed using the Improm-II^TM^ Reverse Transcription system (Promega) following manufacturer’s instructions in which 20 μl of reaction mixture contained 2 μg of total RNA, 0.2 μg random hexamers,1x ImProm-II™ reaction buffer, 3 mM MgCl_2_, 0.5 mM dNTP mix and 1 μl of ImProm-II™ reverse transcriptase. The reaction was incubated at 25°C for 5 min before shifting to 42°C for 60 min and terminated at 70°C for 15 min. The cDNA was stored at -20°C until used.

Quantitative gene expression analysis was performed using an Mx3000P^TM^ Real-Time PCR System. Each amplification reaction was performed in a 12 μl of reaction mixture containing 1x Master mix (KAPA^TM^SYBR^®^Fast), 0.2 μM each of primer, 2 μl of cDNA. Thermal profiles consisted of an initial denaturation at 95°C for 5 min followed by 40 cycles of denaturation at 95°C for 30 sec, annealing at 60°C for 30 sec and extension at 72°C for 30 sec and dissociation at 95°C for 1 min, 55°C for 30 sec and 95°C for 30 sec. Expression of 18s rRNA gene was used to normalize the expression of FBP1. Expression data were calculated from the cycle threshold (Ct) value using the ΔCt method of quantification. Oligonucleotides used for qPCR are listed in Table 1.

### Western blot analysis

50 μg of nuclear proteins were separated by 10% discontinuous SDS–PAGE under reducing conditions [33]. Proteins were transferred to a PVDF membrane using semi-dry blotting and subjected to immunological detection. The C/EBPα band was detected by rabbit anti-C/EBPα polyclonal antibody while the HNF4α band was detected by anti-HNF4α polyclonal antibody. Anti-β-actin monoclonal antibody was used for normalizing protein loading. The immunoreactive bands were visualized upon adding mouse anti-rabbit IgG or ratbbit anti-mouse IgG secondary antibodies followed using the enhanced chemiluminescence detection system (Pierce).

### Statistical analysis

All data are presented as the means ± SD from three independent experiments. Statistical significance between samples was determined by using one way analysis of variance, Sigma Stat 3.5.

## Results

### Induction of human FBP1 expression by C/EBPα and HNF4α in HepG2 cells

**Fig 1:**
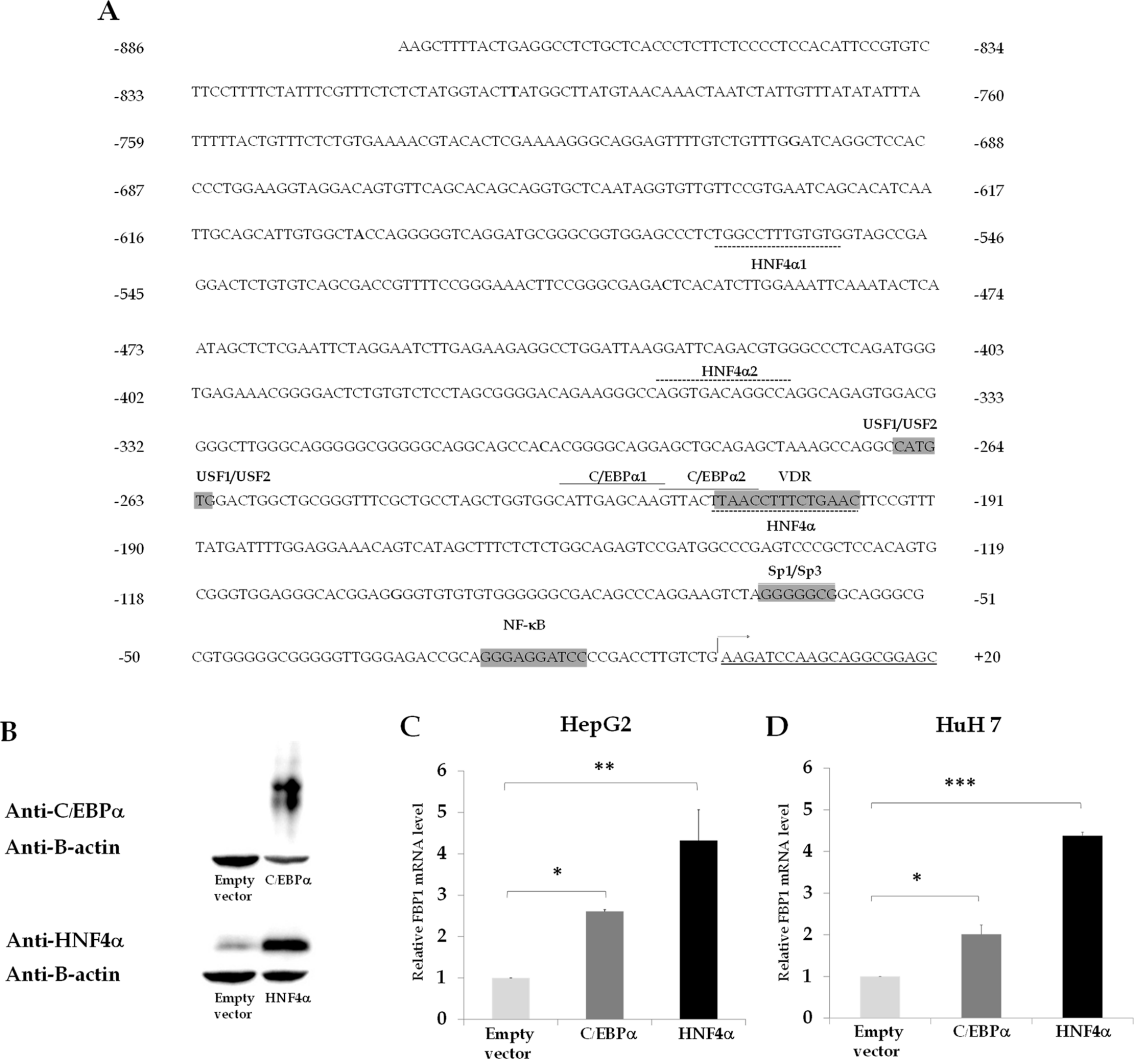
Human FBP1 gene promoter and induction of hFBP1 expression in HepG2 by C/EBPα or HNF4α. (A) Nucleotide sequence of human FBP1 promoter with various putative transcription factor binding sites identified by PROMO [34] and JASPAR databases [35]. The binding sites of previously identified transcription factors including Sp1, USFs, NF-κB [31] and VDRE [36, 37] are also included. (B) Western blot analysis of HepG2 cells transfected with pcDNA3 (empty vector), pcDNA3-C/EBPα or pcDNA3-HNF4α using anti-C/EBPα or anti-HNF4α antibodies. Control loading was assessed by probing the blot with anti-β-actin antibody. (C) qRT-PCR of FBP1 expression of HepG2 cells transfected with empty vector, C/EBPα or HNF4α. Human FBP1 mRNA expression was normalized with that of 18s rRNA, and shown as relative expression. (D) qRT-PCR of FBP1 expression in Huh7 cells transfected with empty vector, C/EBPα or HNF4α. The values obtained from cells transfected with pcDNA3-C/EBPα or pcDNA3-HNF4α were relative to that obtained from cells transfected with empty vector, which was arbitrarily set as 1. The values shown are means ± standard deviation of three independent experiments (n = 3). The statistical analysis was conducted by ANNOVA test where *p < 0.01 and **p < 0.05.

A previous study identified some ubiquitous transcription factors such as Sp1, USF1, USF2 and NF-κB that bind to their cognate sequences (Fig 1A) located in the proximal region of the human FBP1 promoter [30]. However, the transcription factors that regulate liver specific energy metabolism were not identified. Using PROMO [34] and JASPAR databases [35], which predict regulatory elements in eukaryotic promoters, we were able to identify two putative binding sites for the CCAAT-enhancer binding protein-α (C/EBPα), located at nucleotides -228/-218 (designated C/EBPα1 site: 5’-ATTGAGCAAG-3’) and -218/-208 (C/EBPα2 site: 5’-GTTACTTAAC-3’), and three binding sites for hepatocyte nuclear factor-4α, located at nucleotides -556/-554 (designated HNF4α1 site: 5’-TGGCCTTTGTGTG-3’: antisense strand), (HNF4α2 site: 5’-AGGTGACAGGCCA-3’: sense strand) and the -212/-198 (HNF4α site3: 5’-TAACCTTTCTGAACT-3’: antisense strand) (Fig 1A).

To examine whether these two transcription factors can regulate FBP1 expression, C/EBPα or HNF4α was overexpressed in hepatocyte HepG2 cells. As shown in Fig 1B, both transcription factors were successfully overexpressed in HepG2, resulting in 3-fold and 4-fold increase in the levels of hFBP1 mRNA expression (Fig 1C), respectively. Similar results were obtained when HNF4α or C/EBP1α was overexpressed in Huh7, another human hepatocyte cell line (Fig 1D). These results indicate that both HNF4α and C/EBPα can act as activators of FBP1 transcription in human hepatocytes.

### C/EBPα regulates expression of human FBP1 through two C/EBPα binding sites

**Fig 2:**
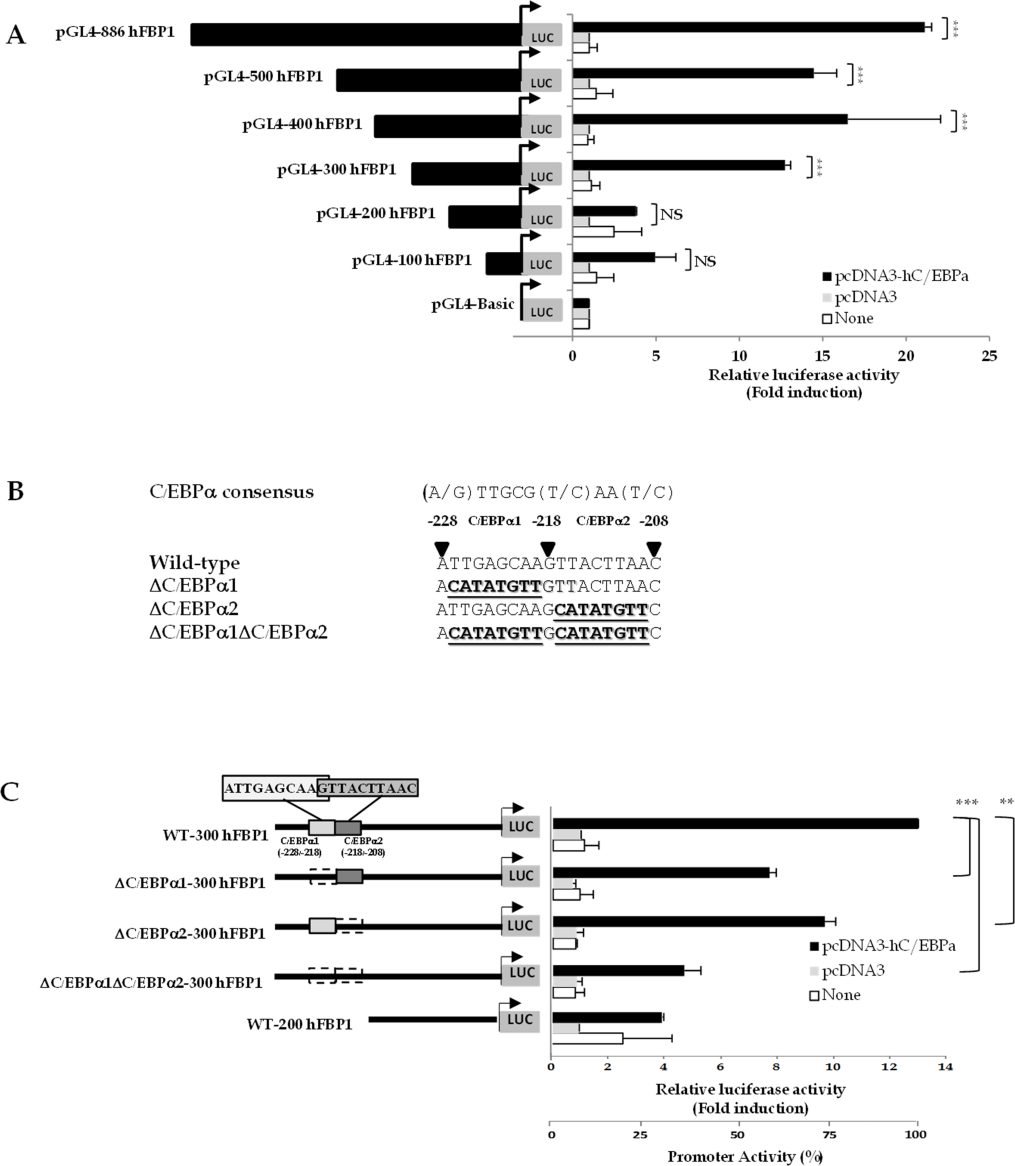
Identification of functional C/EBPα binding sites in human FBP1 promoter. (A) Transactivation of 5’-truncated hFBP1 promoter-luciferase reporter construct by C/EBPα in HepG2 cells. The 886 nucleotides-hFBP1 promoter-luciferase reporter gene or its 5’-truncated constructs (500, 400, 300, 220 and 100 nucleotides) were transiently co-transfected with empty vector (pcDNA3; grey bar) or plasmid overexpressing C/EBPα (pcDNA3-C/EBPα; black bar) into HepG2. The luciferase activity of wild type or mutant construct was normalized with β-galactosidase activity shown as relative luciferase activity. Relative luciferase activity obtained from the cells transfected with the hFBP1-promoter-luciferase constructs and plasmid encoding C/EBPα protein was presented as “fold change” relative to those transfected with hFBP1-promoter-luciferase construct and empty vector, which was arbitrarily set as 1. (B) Nucleotide sequences of two overlapping C/EBPα binding designated C/EBPα1 (-227/-218) and C/EBPα2 (-218/-209) in hFBP1 promoter and its mutagenic sequences in ΔC/EBPα1 and ΔC/EBPα2 or double mutant ΔC/EBPα1 ΔC/EBPα2. Underline indicates nucleotide changes of each mutant. (C) Effect of mutations of two overlapping C/EBPα binding sites on C/EBPα transactivation of FBP1 promoter activity. Single or double mutations of C/EBPα1 and C/EBPα2 sites in 300 hFBP1 promoter-reporter construct and co-transfected with empty vector (pcDNA3; grey bar) or vector containing C/EBPα (pcDNA3-C/EBPα; black bar) into HepG2 cells. The luciferase activity of each construct was normalized to β-galactosidase activity and expressed as relative luciferase activity. Relative luciferase activity obtained from cells transfected with WT or mutated FBP1 promoter-luciferase and plasmid overexpressing C/EBPα was presented as fold change relative to those transfected with the parental or mutated FBP1-luciferase reporter and pcDNA empty vector, which was arbitrarily set as 1. The values shown are means ± standard deviation of three independent experiments (n = 3). The statistical analysis was conducted by ANNOVA test where *p < 0.01, **p < 0.05, ***p < 0.001.

To further investigate whether this positive effect on FBP1 expression is mediated through the above C/EBPα binding sites, we performed transactivation assays in which the -886 human FBP1 promoter-luciferase reporter construct containing the first 886 nucleotides of hFBP1 promoter (pGL4-886hFBP1) was co-transfected with plasmid overexpressing C/EBPα into HepG2 cells. As shown in Fig 2A, overexpression of C/EBPα resulted in a 20-fold increase in the luciferase activity from the FBP1 promoter-luciferase reporter construct. Truncations of the upstream sequences of FBP1 promoter to nucleotide positions -500 (pGL4-500hFBP1), -400 (pGL4-400hFBP1) and -300 (pGL4-300hFBP1) resulted in the further reduction of C/EBPα-mediated activation of luciferase activity to about 12-15- fold. However, further deletion of the 5’-end to nucleotide positions -200 (pGL4-200hFBP1) and -100 (pGL4-100hFBP1) resulted in only 3-fold induction by C/EBPα, suggesting that the major determinant for C/EBPα response is located between nucleotides -300 and -200, corresponding to the two putative C/EBPα sites at -228/-218 (C/EBPα1) and -218/-208 (C/EBPα2). To further examine which of these two sites confers the C/EBPα response, we mutated C/EBPα site 1 or 2 to unrelated sequences (Fig 2B) in the pGL4-300hFBP1 construct that contains these two C/EBPα sites and co-transfected them with a plasmid encoding C/EBPα. As shown in Fig 2C, mutations of the C/EBPα1 (pGL4-ΔC/EBPα1) or C/EBPα2 (pGL4-ΔC/EBPα2) decreased the promoter activity by 40% and 25%, respectively, while double mutation of both sites (ΔC/EBPα1ΔC/EBPα2) further reduced the promoter activity by 65%. The marked reduction of C/EBPα response in the double mutation construct was also similar to the pGL4-200hFBP1 that lacks both C/EBPα binding sites, suggesting that both C/EBPα1 and C/EBPα2 sites act cooperately to maximize FBP1 expression in HepG2 cells.

**Fig 3:**
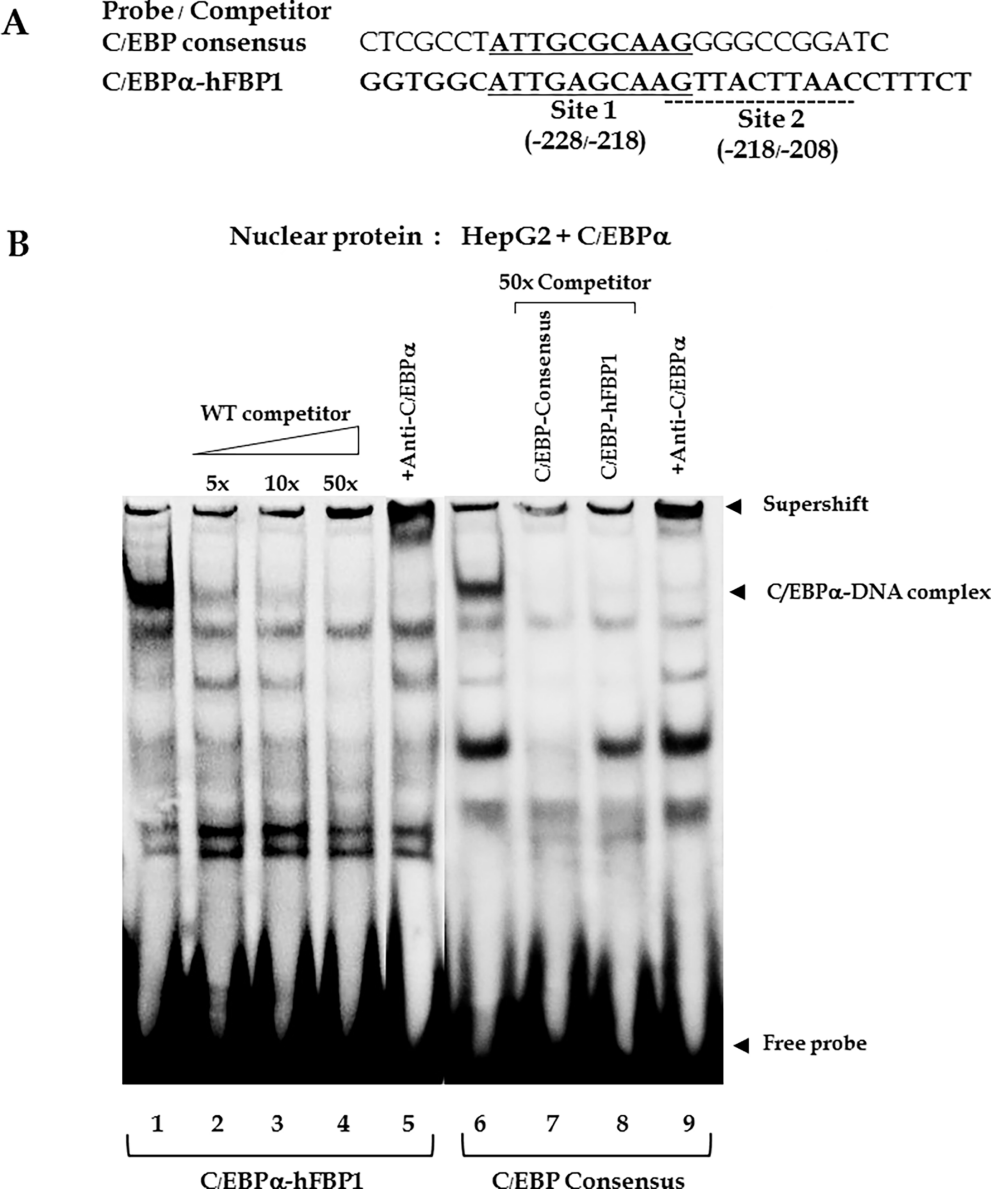
EMSA of C/EBPα binding site in humanFBP1 promoter. (A) Oligonucleotide sequences of C/EBPα consensus binding site and two overlapping C/EBPα binding sites (-227/-218 and -218/-209) in hFBP1promoter used in EMSA. Underline shows the core binding site. (B) EMSA of -227/-218 and -218/-209 C/EBPα binding site of FBP1 promoter comparing with consensus C/EBPα binding site or C/EBPα-hFBP1 site. Biotin-labeled double stranded containing two overlapping C/EBPα binding sites of FBP1 with nuclear extract of HepG2 cells overexpressing C/EBPα (lanes 1 and 5), together with 5x, 10x, 50x excess unlabeled C/EBPα-FBP1 probe (lanes 2–4), or with xx unlabeled consensus C/EBPα probe (lane 6) or with anti-C/EBPα antibody (lane 7). Biotin-labeled C/EBPα consensus sequence was also used as a positive control of which this probe was incubated with nuclear extract of HepG2 cells overexpressing C/EBPα alone (lane 8) or together with unlabeled consensus C/EBPα (lane 9), or unlabeled C/EBPα-FBP1 (lane 10) or with anti-C/EBPα antibody (lane 11).

To confirm whether C/EBPα indeed binds to any of these two C/EBPα sites, EMSA was performed using double stranded oligonucleotide spanning these two sites (C/EBPα-hFBP1), compared with a consensus C/EBPα binding site [38] (see sequence in Fig 3A) and a nuclear extract of HepG2 cells overexpressing C/EBPα. As shown in Fig 3B, the FBP1 probe harboring both C/EBPα sites produced a predominant DNA-protein complex (lane 1). Addition of 5x, 10x and 50x unlabeled C/EBPα-FBP1 probe gradually decreased the complex formation. Incubation of the binding reaction in the presence of anti-C/EBPα antibody markedly prevented the complex formation concomitant with the formation of a supershifted band (lane 5). A similar pattern of DNA-protein binding was observed when the consensus C/EBPα probe was incubated with the nuclear extract of HepG2 cells overexpressing C/EBPα (lane 6). Similar to C/EBPα -FBP1 probe, addition of an unlabeled consensus C/EBPα probe or the C/EBPα -FBP 1 probe (lanes 7 and 8) in the binding reaction eliminated the formation of DNA-protein complex and addition of anti-C/EBPα antibody produced a supershifted band (lane 9).

### HNF4α regulates expression of human FBP1 through an HNF4-specific binding motif (H4SBM) and direct repeat DR3

**Fig 4:**
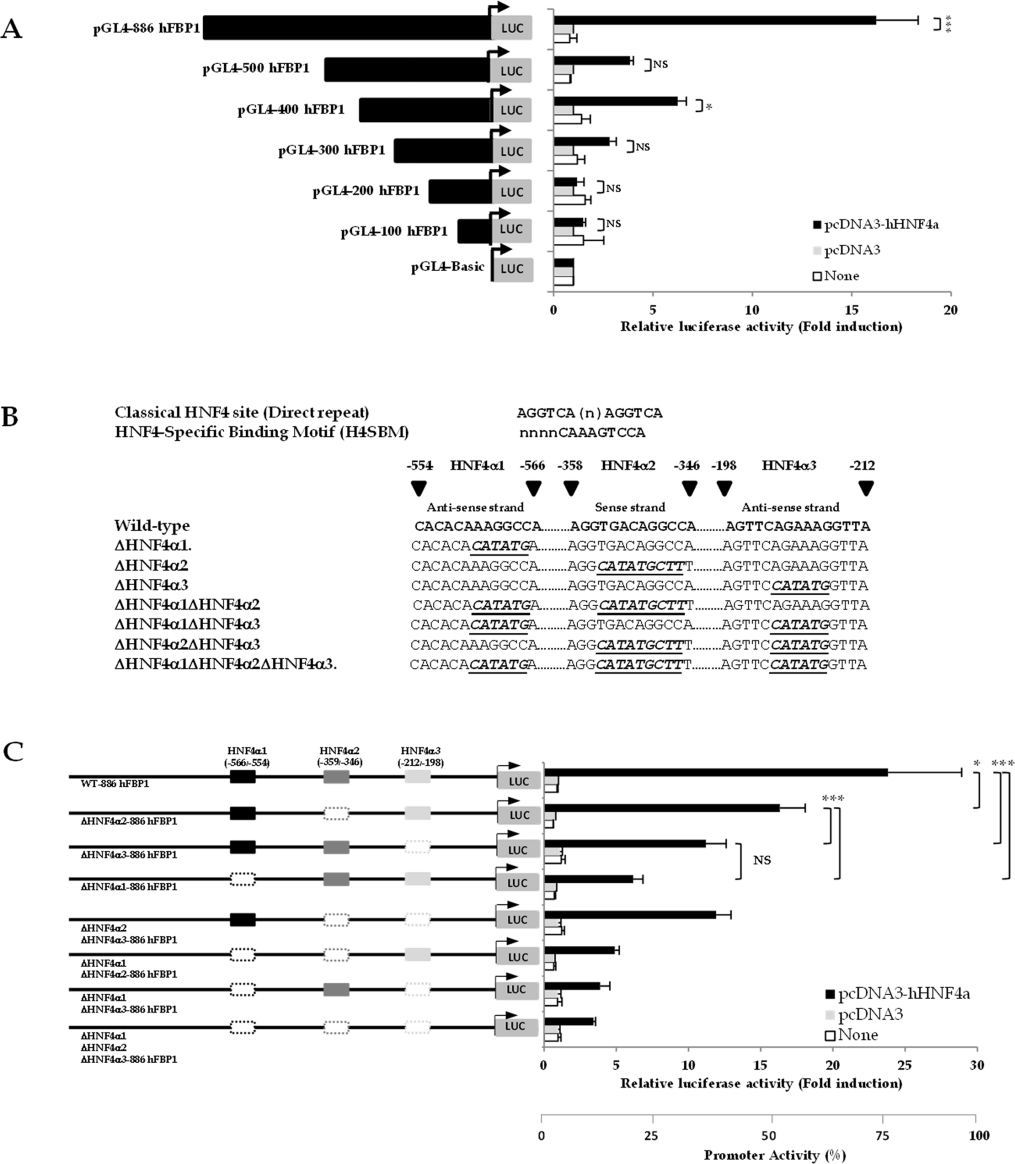
Identification of HNF4α binding sites in hFBP1 promoter. (A) Transactivation of 5’-truncated hFBP1 promoter-luciferase reporter construct by HNF4α in HepG2 cells. The 886 nucleotides-hFBP1 promoter-luciferase reporter gene or its 5’-truncated mutants (500, 400, 300, 200 and 100) were transiently co-transfected with empty vector (pcDNA3; grey bar) or plasmid overexpressing HNF4α (pcDNA3-HNF4α; black bar) into HepG2. The luciferase activity of wild type or mutant construct was normalized with β-galactosidase activity and shown as relative luciferase activity. Relative luciferase activity obtained from cells co-transfected with the hFBP1-promoter-luciferase constructs and plasmid encoding HNF4α was presented as “fold change” relative to those transfected with hFBP1-promoter-luciferase construct and empty vector, which was arbitrarily set as 1. (B) Nucleotide sequences of consensus HNF4α binding sites including classical DR1 and H4-SBM and three HNF4α binding sites (HNF4α1 (-566/-554, antisense strand), HNF4α2 (-358/-346; sense strand) and HNF4α3 site (-212/-198; antisense strand)) in hFBP1 promoter also shown (C) Effect of mutations of three HNF4α binding sites on FBP1 promoter activity. Mutations of the HNF4α1, HNF4α2 and HNF4α3 sites were introduced in the 886 FBP1 promoter-reporter construct and co-transfected with empty vector (pcDNA3; grey bars) or vector containing human HNF4α (pcDNA3-HNF4α; black bars) into HepG2 cells. The luciferase activity of each construct was normalized to β-galactosidase activity and expressed as relative luciferase activity. The values obtained from mutated constructs are expressed relative to the corresponding parental or mutant construct which was arbitrarily set as 100%. The values shown are means ± standard deviation (n = 3). *p < 0.01, **p < 0.05, ***p <0.001.

We next investigated whether the stimulatory effect of HNF4α on endogenous FBP1 expression is mediated through the three putative HNF4α binding sites shown in Fig 2. We performed 5’- truncation analysis of the FBP1 promoter to localize the *cis*-acting element(s) that mediates HNF4α activation. The same set of 5’-truncated FBP1 promoter mutant constructs that were used to identify C/EBPα binding sites were co-transfected with a plasmid overexpressing HNF4α. As shown in Fig 4A, truncation of the FBP1 promoter from nucleotide positions -886 to -500 resulted in a marked reduction of luciferase activity (65%), indicating the presence of the first HNF4α-responsive sequence between these nucleotides. However, further deletion to -400 resulted in a slight increase of the reporter activity while further deletion to -300 resulted in 60% reduction of luciferase activity, suggesting the presence of a second HNF4α-responsive element. Likewise, further truncation to -200 lowered the reporter activity by 50%, indicating the presence of the third HNF4α-responsive element, locating between nucleotides -300 to -200. The presence of three HNF4α-responsive regions between nucleotides -856 to -500, -400 to -300 and -300 to -100 is consistent with the three HNF4α binding sites shown in Fig 1. It is noted that the -556/-554 HNF4α (HNF4α1 site: 5’-TGGCCTTTGTGTG-3’: antisense strand) resembles the HNF4-specific binding motif (H4SBM: 5’-NNNNCAAAGTCCA-3’) as described by Fang *et al*., 2012 [39] except it contains one nucleotide divergent from the H4SBM consensus sequence (underlined). In contrast, the -359/-346 (HNF4α2 site: 5’-AGGTGAcAGGCCA-3’: sense strand) and the -212/-198 (HNF4α site3: 5’-TAACCTttcTGAACT-3’: antisense strand) are similar to the classical direct repeat 1 (DR1) and DR3, respectively for tor the nuclear receptor [5’-AGGTCA(N)_1-__3_AGGTCA-3’] for the nuclear receptors (NR) [40]. However, both HNF4α2 and HNF4α3 sites contain two nucleotides different (underlined) from the DR motif. We next confirmed the functional importance of these sites by mutating them to the sequences shown in Fig 4B and used them for the transactivation assay with HNF4α. As shown in Fig 4C, single mutation of HNF4α1, HNF4α2 and HNF4α3 site decreased HNF4α mediated transactivation of FBP1 promoter activity by 75%, 35% and 50%, respectively. Mutations of HNF4α1 together with HNF4α2 or HNF4α3 decreased the reporter activity that was similar to the single HNF4α1 mutation while double mutation of HNF4α2 and HNF4α3 sites lowered reporter activity by 50%. Mutations of three HNF4α binding sites resulted in 80% reduction of HNF4α-mediated transactivation activity. These results indicated that the functional importance of HNF4α binding sites with respect to HNF4α- transactivation activity being HNF4α1 > HNF4α3 > HNF4α2, respectively.

**Fig 5:**
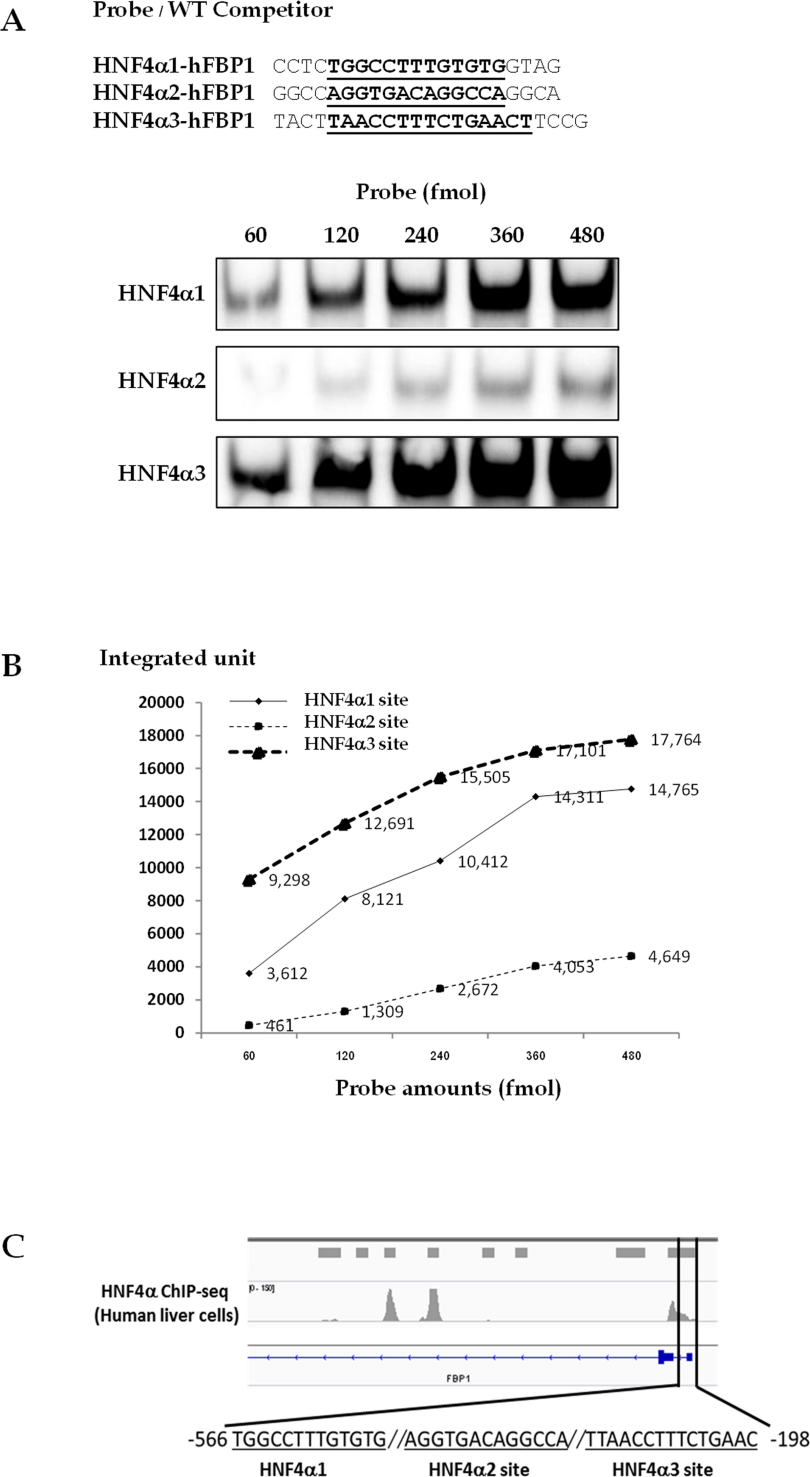
Interaction of HNF4α to three HNF4α binding sites in human FBP1 promoter. **(A)** Biotin-labelled double stranded nucleotides corresponding to three HNF4α binding sites in the hFBP1 promoter used for EMSA. The core HNF4α binding sites in each probe are highlighted. (B) EMSA of various amounts of FBP1 probes (60, 120, 240, 360 and 280 fmole) harboring different HNF4α binding site in the presence of 200 ng of purified HNF4α. (C) The intensities of the HNF4α-bound complexes were plotted against the amounts of each probe. (D) HNF4α binding site in FBP1 promoter mapped by ChIP seq in human adult liver cell.

To confirm binding of HNF4α to these three sites, electrophoretic mobility shift assays (EMSA) were performed by incubating various concentration of oligonucleotide probes harboring HNF4α1, HNF4α2 or HNF4α3 sites (60, 120, 240, 360 and 480 fmol) with a limited amount (200 ng) of purified hHNF4α (Fig 5A). Quantification of the HNF4α-DNA complex bands in Fig 5B demonstrated that HNF4α bound to site 3 with slightly higher affinity than to site 1 while its affinity for site 2 was much lower. Although HNF4α binds to HNF4α1 and HNF4α3 sites with slightly different affinities, it seems that both sites are important for HNF4α-mediated activation of hFBP1 promoter activity as indicated by mutation of either sites produced a great effect on expression of the reporter gene in Fig 4. Conversely, this EMSA showed the poor binding of HNF4α to the HNF4α2 site, suggesting that HNF4α2 site is the insignificant binding site for HNF4α. Analysis of chromatin immunoprecipitation sequence (ChIP seq) of HNF4α in genomic DNA of adult liver cells mapped by ENCODE project [41] reveals the presence of one broad peak covering the transcription start site to the first 600 nucleotides upstream of FBP1 gene which includes HNF4α1(-566/-554, antisense strand), HNF4α2 (-358/-346) and HNF4α3 (-212/-198) (Fig 5C) sites, confirming the prediction by JASPAR prediction, reporter assay and EMSA.

### siRNA-suppression of HNF4α and C/EBPα lowered expression of FBP1 and other gluconeogenic enzymes

**Fig 6:**
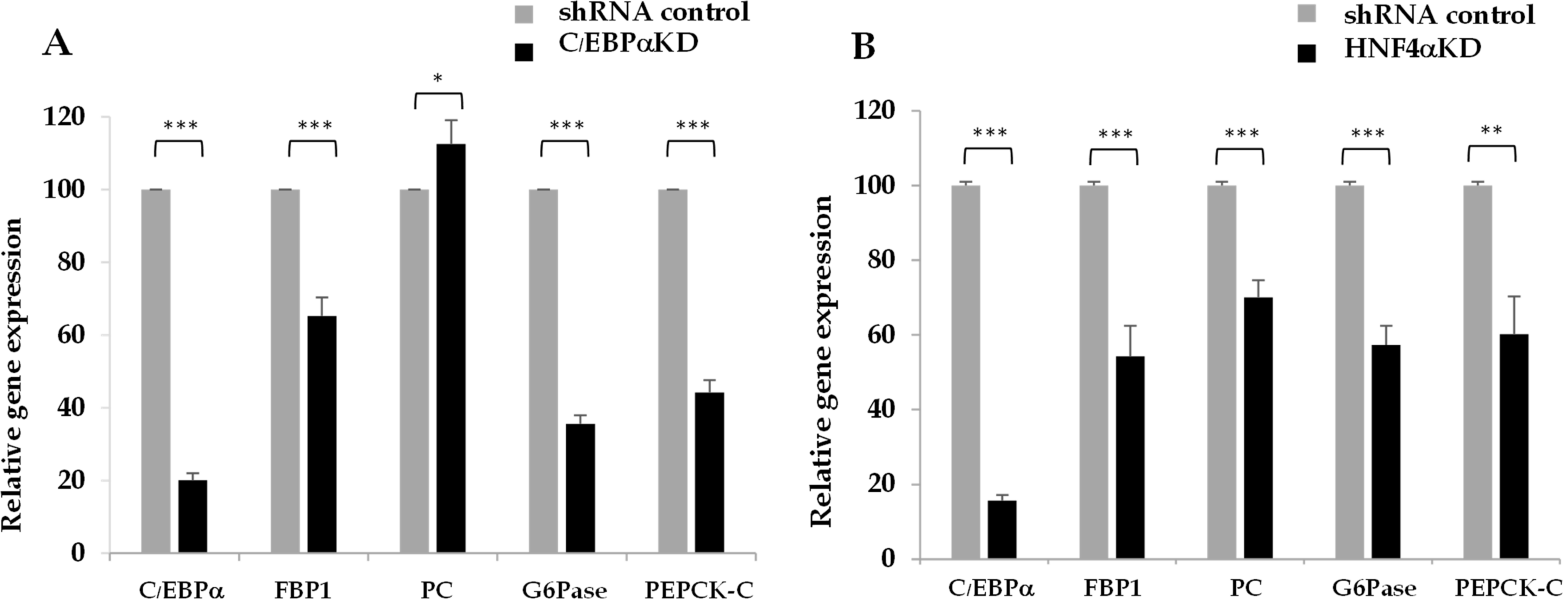
Suppression of C/EBPα or HNF4α lowered expression of gluconeogenic enzyme mRNAs. (A) Suppression of C/EBPα siRNA (B) Suppression of HNF4α siRNA. The expression of PC, PEPCK-C, FBP1 and G6PC1 was measured by quantitative real time PCR and normalized to that of 18s rRNA. The values obtained from C/EBPα or HNF4α knockdown HepG2 cells is expressed relative to that obtained from the scramble control group (shRNA control) which was arbitrarily set as 100%. Statistical analysis was performed by ANNOVA test where *p < 0.01, **p < 0.05, ***p < 0.001.

We next asked whether down-regulation of C/EBPα or HNF4α expression would affect expression of FBP1 expression in HepG2 cells. HepG2 cells were transiently transfected with siRNAs targeted to C/EBPα and HNF4α and the expression of FBP1 and other gluconeogenic enzymes including PC, PEPCK-C, and G6PaseI were measured by qPCR. As shown in Fig 6, knocking down of C/EBPα expression by 80% resulted in 35%, 55% and 65% reduction of FBP1, PEPCK-C and G6PaseI mRNA expression, respectively while minimally affected expression of PC mRNA. In contrast, siRNA-mediated suppression of HNF4α resulted in simultaneous down-regulation of FBP1, PC, PEPCK-C and G6Pase1 by 45%, 30%, 60% and 40%, respectively. These data indicate that HNF4α have a strong influence on regulation of all four gluconeogenic enzymes while C/EBPα also regulates expression of most gluconeogenic enzymes except PC.

## Discussion

Our result shows for the first time that human FBP1 is regulated by two important transcription factors, C/EBPα and HNF4α. C/EBPα is a member of the basic region leucine zipper family that binds to a sequence motif 5’-(A/G)TTGCG(C/T)AA(C/T)-3’ [38]. C/EBPα regulates transcription by forming a complex with two transcription co-activators, p300 or CBP and enhances RNA polymerase II basal transcriptional activity [42, 43]. Regarding its targets, C/EBPα regulates transcription of genes whose products are involved in development and central metabolism [44, 45]. Since C/EBPα expression is most abundant in liver, abrogation of its expression severely affected hepatocyte development and metabolism [46, 47]. Previous studies show that null or liver-specific C/EBPα knockout mice develop severe fasting hypoglycemia due to impaired postnatal gluconeogenesis accompanied by a marked reduction of expression of PEPCK-C and G6Pase1 but no data were available regarding regulatory role of C/EBPα on other gluconeogenic enzymes [47, 48]. The only evidence that shows that C/EBPα may be involved in regulation of FBP1 expression come from studies by He et al. [49, 50] demonstrating that loss of function of CBP, a transcription co-activator of C/EBPα resulted in impaired fasting-induced gluconeogenesis accompanied with reduced expression of PEPCK-C, FBP1 and G6Pase, underscoring the regulatory role of this transcription factor in controlling gluconeogenesis. Here we show that overexpression of C/EBPα results in up-regulation of endogenous FBP1 in HepG2 cells. This transcriptional activation is mediated through the two overlapping C/EBPα binding sites, located at nucleotides -227/-218 (C/EBPα1, 5’-ATTGAGCAAG-3’) and -218/-209 (C/EBPα2, 5’-GTTACTTAAC-3’). Both of which contain two nucleotides (underlined) different from the consensus sequence [(A/G)TTGCG(T/C)AA(T/C)] [38]. These two overlapping C/EBPα binding sites appear to work in concert because mutating either one of these two sites produced only marginal or moderate reduction of C/EBPα-mediated transcriptional activation of the hFBP1 promoter while double mutation produced a more pronounced effect. EMSA using an hFBP1 probe containing these C/EBPα sites with a nuclear extract of HepG2 cells overexpressing C/EBPα clearly confirmed its binding to the these two C/EBPα binding sites. Although we were able to show that C/EBPα binds to these two overlapping C/EBPα sites by EMSA, we could not detect *in situ* interaction of C/EBPα in HepG2 cells using ChiP assay. This failure may suggest a relative poor binding of C/EBPα to its cognate sequence in FBP1 promoter *in vivo*. Nevertheless we were able to show that down-regulation of C/EBPα by siRNA produces a great impact on the expression of FBP1 together with other gluconeognic enzymes except PC. Collectively, our data demonstrate that C/EBPα plays an important role in programming the gluconeogenic pathway in human liver through PEPCK-C, FBP1 and G6Pase1. It is noted that C/EBPα expression is also under hormonal control. Rat hepatoma cells treated with dexamethasone or cAMP show a marked increase in the expression of C/EBPα mRNA while insulin opposes this effect [51]. It would be obvious that during caloric deprivation, gluconeogenic enzymes would be upregulated via cAMP-induced C/EBPα expression. This is also observed in the liver of the C/EBPα knockout mouse that showed impaired cAMP-induced PEPCK-C transcription [52].

HNF4α is a liver-enriched transcription factor, belonging to member of hormone nuclear superfamily and highly expressed in liver as is C/EBPα [53]. In liver, HNF4α plays a role in regulating expression of several genes involved in metabolic pathways including carbohydrate, lipid and bile acid metabolism [54]. Mutations of HNF4α gene in humans cause maturity onset diabetes of the young 1 (MODY1) [55]. Here we showed that human FBP1 expression is regulated by HNF4α as in PC, PEPCK-C and G6PaseI [31, 56, 57]. Overexpression of this transcription factor in HepG2 cells resulted in 4-fold increase in FBP1 mRNA expression. This transcriptional activation by HNF4α is mediated through three HNF4α binding sites in the FBP1 promoter, located at -556/-554 (HNF4α1), -359/-346 (HNF4α2) and the -212/-198 (HNF4α3). HNF4α1 resembles the H4-SBM which is specific for binding by HNF4α while HNF4α2 and HNF4α3 resemble the classical direct repeat, binding site for several nuclear receptors. Mutational analysis demonstrates that these three sites confer differential responses to HNF4α transactivation being site 1 > site 3 > site 2, respectively. The use of these three HNF4α binding sites for HNF4α transactivation of FBP1 expression was also confirmed by HNF4α-ChiP seq data of adult liver cells demonstrating binding of HNF4α across these three binding sites.

HNF4α has previously been reported as the vitamin D3-responsive element (VDRE)/retinoic acid responsive element (RAR) that mediates vitamin-D/retinoic acid–induced FBP1 expression in monocytes [36, 37]. It is possible that vitamin D receptor and HNF4α may share the same responsive element for transcription activation of FBP1 expression in monocytes and hepatocytes, respectively. Sharing a common responsive element for mediating transcriptional activation by two distinct nuclear receptors in different tissues is not uncommon [58, 59]. One example is for PC, one of the four gluconeogenic enzymes in which its promoter contains DR1 that enables HNF4α or PPARγ to bind to and mediate its expression in hepatocytes and adipocytes, respectively [31, 60]. Regulation of FBP1 expression by HNF4α is probably associated with the peroxisome proliferator activated receptor 1α (PGC1α) that plays a key role in coordinating gluconeogenic enzyme levels in liver. Previous studies showed that fasting-induced PGC1α expression or ectopic expression of PGC1α in primary rat or mouse hepatocytes induced expression of PEPCK-C, G6Pase1 and FBP1, concomitant with increased hepatic gluconeogenesis [61, 62]. This transcriptional activation of PGC1α on gluconeogenic genes is mediated through the physical interaction with HNF4α [63]. Our finding that human FBP1 expression is regulated by HNF4α provides a further link between PGC1α and FBP1 expression during fasting. Lastly, we showed that suppression of HNF4α expression in HepG2 cells resulted in a simultaneous down-regulation of all gluconeogenic enzymes similar to what is observed in rodents models in which ablation of this transcription factor *in vivo* or in cultured hepatocytes also affect expression of all gluconeogenic enzymes [31, 64]

In summary we show that C/EBPα and HNF4α are the two important transcription factors that regulate expression of human FBP1 expression in HepG2 cells. This transcriptional activation is mediated through binding to -227/-218 and -218/-209 C/EBPα binding sites and through -566/-554, and -212/-198 HNF4α binding sites, respectively. Suppression of expression of both transcription factors results in a marked decrease in expression of all gluconeogenic enzymes.

## References

[1] Klover PJ, Mooney RA. Hepatocytes: critical for glucose homeostasis. Int J Biochem Cell Biol. 2004; 36(5):753–8. 15061128 10.1016/j.biocel.2003.10.002

[2] Petersen MC, Vatner DF, Shulman GI. Regulation of hepatic glucose metabolism in health and disease. Nat Rev Endocrinol. 2017; 13(10):572–87. doi: 10.1038/nrendo.2017.80 28731034 10.1038/nrendo.2017.80 PMC5777172

[3] Barthel A, Schmoll D. Novel concepts in insulin regulation of hepatic gluconeogenesis. Am J Physiol Endocrinol Metab. 2003; 285(4):E685–92. doi: 10.1152/ajpendo.00253.2003 12959935 10.1152/ajpendo.00253.2003

[4] Sharabi K, Tavares CD, Rines AK, Puigserver P. Molecular pathophysiology of hepatic glucose production. Mol Aspects Med. 2015; 46:21–33. doi: 10.1016/j.mam.2015.09.003 26549348 10.1016/j.mam.2015.09.003 PMC4674831

[5] Nordlie RC, Foster JD, Lange AJ. Regulation of glucose production by the liver. Annu Rev Nutr. 1999; 19:379–406. doi: 10.1146/annurev.nutr.19.1.379 10448530 10.1146/annurev.nutr.19.1.379

[6] Tejwani GA. Regulation of fructose-bisphosphatase activity. Adv Enzymol Relat Areas Mol Biol. 1983; 54:121–94. 6303063 10.1002/9780470122990.ch3

[7] Schaftingen EV, Hers HG. Inhibition of fructose-1,6-bisphosphatase by fructose-2,6-bisphosphate. Proc Natl Acad Sci USA. 1981; 78(5):2861–3. 6265919 10.1073/pnas.78.5.2861 PMC319458

[8] El-Maghrabi MR, Pilkis SJ. Rat liver 6-phosphofructo 2-kinase/fructose 2,6-bisphosphatase: a review of relationships between the two activities of the enzyme. J Cell Biochem. 1984; 26(1):1–17. doi: 10.1002/jcb.240260102 6096384 10.1002/jcb.240260102

[9] Tillmann H, Eschrich K. Isolation and characterization of an allelic cDNA for human muscle fructose-1,6-bisphosphatase. Gene. 1998; 212(2):295–304. 9678974 10.1016/s0378-1119(98)00181-4

[10] Tillmann H, Stein S, Liehr T, Eschrich K. Structure and chromosomal localization of the human and mouse muscle fructose-1,6-bisphosphatase genes. Gene. 2000; 247(1–2):241–53. 10773464 10.1016/s0378-1119(00)00079-2

[11] Rakus D, Pasek M, Krotkiewski H, Dzugaj A. Interaction between muscle aldolase and muscle fructose 1,6-bisphosphatase results in the substrate channeling. Biochemistry. 2004; 43(47):14948–57. doi: 10.1021/bi048886x 15554702 10.1021/bi048886x

[12] Rakus D, Gizak A, Kasprzak AA, Zarzycki M, Maciaszczyk-Dziubinska E, Dzugaj A. The mechanism of calcium-induced inhibition of muscle fructose 1,6-bisphosphatase and destabilization of glyconeogenic complex. PLoS One. 2013; 8(10):e76669 doi: 10.1371/journal.pone.0076669 24146906 10.1371/journal.pone.0076669 PMC3795747

[13] Andrikopoulos S, Rosella G, Gaskin E, Thorburn A, Kaczmarczyk S, Zajac JD, et al Impaired regulation of hepatic fructose-1,6-bisphosphatase in the New Zealand obese mouse model of NIDDM. Diabetes. 1993; 42(12):1731–6. 8243819 10.2337/diab.42.12.1731

[14] Andrikopoulos S, Rosella G, Kaczmarczyk SJ, Zajac JD, Proietto J. Impaired regulation of hepatic fructose-1,6-biphosphatase in the New Zealand Obese mouse: an acquired defect. Metabolism. 1996; 45(5):622–6. 8622607 10.1016/s0026-0495(96)90034-7

[15] Wu C, Okar DA, Newgard CB, Lange AJ. Overexpression of 6-phosphofructo-2-kinase/fructose-2, 6-bisphosphatase in mouse liver lowers blood glucose by suppressing hepatic glucose production. J Clin Invest. 2001; 107(1):91–8. doi: 10.1172/JCI11103 11134184 10.1172/JCI11103 PMC198549

[16] Visinoni S, Fam BC, Blair A, Rantzau C, Lamont BJ, Bouwman R, et al Increased glucose production in mice overexpressing human fructose-1,6-bisphosphatase in the liver. Am J Physiol Endocrinol Metab. 2008; 295(5):E1132–41. doi: 10.1152/ajpendo.90552.2008 18780768 10.1152/ajpendo.90552.2008

[17] Bertinat R, Pontigo JP, Pérez M, Concha II, San Martín R, Guinovart JJ, et al Nuclear accumulation of fructose 1,6-bisphosphatase is impaired in diabetic rat liver. J Cell Biochem. 2012; 113(3):848–56. doi: 10.1002/jcb.23413 22021109 10.1002/jcb.23413

[18] Samuel VT, Beddow SA, Iwasaki T, Zhang XM, Chu X, Still CD, et al Fasting hyperglycemia is not associated with increased expression of PEPCK or G6Pc in patients with type 2 diabetes. Proc Natl Acad Sci USA. 2009; 106(29):12121–6. doi: 10.1073/pnas.0812547106 19587243 10.1073/pnas.0812547106 PMC2707270

[19] Chen M, Zhang J, Li N, Qian Z, Zhu M, Li Q, et al Promoter hypermethylation mediated downregulation of FBP1 in human hepatocellular carcinoma and colon cancer. PLoS One. 2011; 6(10):e25564 doi: 10.1371/journal.pone.0025564 22039417 10.1371/journal.pone.0025564 PMC3198434

[20] Dong C, Yuan T, Wu Y, Wang Y, Fan TW, Miriyala S, et al Loss of FBP1 by Snail-mediated repression provides metabolic advantages in basal-like breast cancer. Cancer Cell. 2013; 23(3):316–31. doi: 10.1016/j.ccr.2013.01.022 23453623 10.1016/j.ccr.2013.01.022 PMC3703516

[21] Li B, Qiu B, Lee DS, Walton ZE, Ochocki JD, Mathew LK, et al Fructose-1,6-bisphosphatase opposes renal carcinoma progression. Nature. 2014; 513(7517):251–5 doi: 10.1038/nature13557 25043030 10.1038/nature13557 PMC4162811

[22] Hirata H, Sugimachi K, Komatsu H, Ueda M, Masuda T, Uchi R, et al Decreased Expression of Fructose-1,6-bisphosphatase Associates with Glucose Metabolism and Tumor Progression in Hepatocellular Carcinoma. Cancer Res. 2016; 76(11):3265–76. doi: 10.1158/0008-5472.CAN-15-2601 27197151 10.1158/0008-5472.CAN-15-2601

[23] Erion MD, van Poelje PD, Dang Q, Kasibhatla SR, Potter SC, Reddy MR, et al MB06322 (CS-917): A potent and selective inhibitor of fructose 1,6-bisphosphatase for controlling gluconeogenesis in type 2 diabetes. Proc Natl Acad Sci USA. 2005; 102(22):7970–7975. doi: 10.1073/pnas.0502983102 15911772 10.1073/pnas.0502983102 PMC1138262

[24] Dang Q, Kasibhatla SR, Reddy KR, Jiang T, Reddy MR, Potter SC, et al Discovery of potent and specific fructose-1,6-bisphosphatase inhibitors and a series of orally-bioavailable phosphoramidase-sensitive prodrugs for the treatment of type 2 diabetes. J Am Chem Soc. 2007; 129(50):15491–15502. doi: 10.1021/ja074871l 18041834 10.1021/ja074871l

[25] Dang Q, Liu Y, Cashion DK, Kasibhatla SR, Jiang T, Taplin F, et al Discovery of a series of phosphonic acid-containing thiazoles and orally bioavailable diamide prodrugs that lower glucose in diabetic animals through inhibition of fructose-1,6-bisphosphatase. J Med Chem. 2011; 54(1):153–65. doi: 10.1021/jm101035x 21126019 10.1021/jm101035x

[26] Yoshida T, Okuno A, Takahashi K, Ogawa J, Hagisawa Y, Kanda S. Contributions of hepatic gluconeogenesis suppression and compensative glycogenolysis on the glucose-lowering effect of CS-917, a fructose 1,6-bisphosphatase inhibitor, in non-obese type 2 diabetes Goto-Kakizaki rats. J Pharmacol Sci. 2011; 115(3):329–35. 21350313 10.1254/jphs.10262fp

[27] Bie J, Liu S, Li Z, Mu Y, Xu B, Shen Z. Discovery of novel indole derivatives as allosteric inhibitors of fructose-1,6-bisphosphatase. Eur J Med Chem. 2015; 90:394–405. doi: 10.1016/j.ejmech.2014.11.049 25461330 10.1016/j.ejmech.2014.11.049

[28] Kubota K, Inaba S, Nakano R, Watanabe M, Sakurai H, Fukushima Y, et al Identification of activating enzymes of a novel FBPase inhibitor prodrug, CS-917. Pharmacol Res Perspect. 2015; 3(3):e00138 doi: 10.1002/prp2.138 26171222 10.1002/prp2.138 PMC4492754

[29] El-Maghrabi MR, Lange AJ, Jiang W, Yamagata K, Stoffel M, Takeda J, et al Human fructose-1,6-bisphosphatase gene (FBP1): exon-intron organization, localization to chromosome bands 9q22.2-q22.3, and mutation screening in subjects with fructose-1,6-bisphosphatase deficiency. Genomics. 1995; 27(3):520–5. 7558035 10.1006/geno.1995.1085

[30] Herzog B, Waltner-law M, Scott DK, Eschrich K, Granner DK. Characterization of the human liver fructose-1,6-bisphosphatase gene promoter. Biochem J. 2000; 351:385–92. 11023824 PMC1221374

[31] Chavalit T, Rojvirat P, Muangsawat S, Jitrapakdee S. Hepatocyte nuclear factor 4α regulates the expression of the murine pyruvate carboxylase gene through the HNF4-specific binding motif in its proximal promoter. Biochim Biophys Acta. 2013; 1829(10):987–99. doi: 10.1016/j.bbagrm.2013.05.001 23665043 10.1016/j.bbagrm.2013.05.001

[32] Landschulz WH, Johnson PF, Adashi EY, Graves BJ, McKnight SL. Isolation of a recombinant copy of the gene encoding C/EBP. Genes Dev. 1988; 2(7):786–800. 2850264 10.1101/gad.2.7.786

[33] Laemmli UK. Cleavage of Structural Proteins during the Assembly of the Head of Bacteriophage T4. Nature. 1970; 227(5259):680–5. 5432063 10.1038/227680a0

[34] Messeguer X, Escudero R, Farré D, Núñez O, Martínez J, Albà MM. PROMO: detection of known transcription regulatory elements using species-tailored searches. Bioinformatics. 2002; 18(2):333–4. 11847087 10.1093/bioinformatics/18.2.333

[35] Khan A, Fornes O, Stigliani A, Gheorghe M, Castro-Mondragon JA, van der Lee R, et al JASPAR 2018: update of the open-access database of transcription factor binding profiles and its web framework. Nucleic Acids Res. 2018; 46(D1):D260–6. doi: 10.1093/nar/gkx1126 29140473 10.1093/nar/gkx1126 PMC5753243

[36] Fujisawa K, Umesono K, Kikawa Y, Shigematsu Y, Taketo A, Mayumi M, et al Identification of a Response Element for Vitamin D3 and Retinoic Acid in the Promoter Region of the Human Fructose-l,6-bisphosphatase Gene. J Biochem. 2000; 127(3):373–82. 10731708 10.1093/oxfordjournals.jbchem.a022618

[37] Solomon DH, Raynal MC, Tejwani GA, Cayre YE. Activation of the fructose 1,6-bisphosphatase gene by 1,25-dihydroxyvitamin D3 during monocytic differentiation. Proc Natl Acad Sci USA. 1988; 85(18):6904–8. 2842796 10.1073/pnas.85.18.6904 PMC282087

[38] Osada S, Yamamoto H, Nishihara T, Imagawa M. DNA binding specificity of the CCAAT/enhancer-binding protein transcription factor family. J Biol Chem. 1996; 271(7):3891–6. 8632009 10.1074/jbc.271.7.3891

[39] Fang B, Mane-Padros D, Bolotin E, Jaing T, Sladek FM. Identification of a binding motif specific to HNF4 by comparative analysis of multiple nuclear receptors. Nucleic Acids. 2012; 40(12):5343–56. 10.1093/nar/gks190 PMC3384313 22383578

[40] Mangelsdof DJ, Thummel C, Beato M, Herrlich P, Schütz G, Umesono K. The nuclear receptor superfamily: The second decade. Cell. 1995; 83(6):835–9. 8521507 10.1016/0092-8674(95)90199-x PMC6159888

[41] Dunham I, Kundaje A, Aldred SF, Collins PJ, Davis CA, Doyle F, et al An integrated encyclopedia of DNA elements in the human genome. Nature. 2012; 489(7414):57–74. doi: 10.1038/nature11247 22955616 10.1038/nature11247 PMC3439153

[42] Kovacs KA, Steinmann M, Magistretti PJ, Halfon O, Cardinaux JR. CCAAT/enhancer-binding protein family members recruit the coactivator CREB-binding protein and trigger its phosphorylation. J Biol Chem. 2003; 278(38):36959–65. doi: 10.1074/jbc.M303147200 12857754 10.1074/jbc.M303147200

[43] Schwartz C, Beck K, Mink S, Schmolke M, Budde B, Wenning D. Recruitment of p300 by C/EBPbeta triggers phosphorylation of p300 and modulates coactivator activity. EMBO J. 2003; 22(4):882–92. doi: 10.1093/emboj/cdg076 12574124 10.1093/emboj/cdg076 PMC145436

[44] Crosson SM, Roesler WJ. Hormonal regulation of the phosphoenolpyruvate carboxykinase gene. Role of specific CCAAT/enhancer-binding protein isoforms. J Biol Chem. 2000; 275(8):5804–9. 10681569 10.1074/jbc.275.8.5804

[45] McKnight SL, Lane MD, Gluecksohn-Waelsch S. Is CCAAT/enhancer-binding protein a central regulator of energy metabolism? Genes Dev. 1989; 3(12B):2021–4. 2697636 10.1101/gad.3.12b.2021

[46] Lee YH, Sauer B, Johnson PF, Gonzalez FJ. Disruption of the c/ebpα gene in adult mouse liver. Mol Cell Biol. 1997; 17(10):6014–22. 9315660 10.1128/mcb.17.10.6014 PMC232450

[47] Wang ND, Finegold MJ, Bradley A, Ou CN, Abdelsayed SV, Wilde MD, et al Impaired energy homeostasis in C/EBPα knockout mice. Science. 1995; 269(5227):1108–12. 7652557 10.1126/science.7652557

[48] Yang J, Croniger CM, Lekstrom-Himes J, Zhang P, Fenyus M, Tenen DG, et al Metabolic response of mice to a postnatal ablation of CCAAT/enhancer-binding protein alpha. J Biol Chem. 2005; 280(46):38689–990. doi: 10.1074/jbc.M503486200 16166091 10.1074/jbc.M503486200

[49] He L, Naik K, Meng S, Cao J, Sidhaye AR, Ma A. Transcriptional co-activator p300 maintains basal hepatic gluconeogenesis. J Biol Chem. 2012; 287(38):32069–77. doi: 10.1074/jbc.M112.385864 22815486 10.1074/jbc.M112.385864 PMC3442537

[50] He L, Cao J, Meng S, Ma A, Radovick S, Wondisford FE. Activation of basal gluconeogenesis by co-activator p300 maintains hepatic glycogen storage. Mol Endocrinol. 2013; 27(8):1322–32. doi: 10.1210/me.2012-1413 23770612 10.1210/me.2012-1413 PMC3725339

[51] Crosson SM, Davies GF, Roesler WJ. Hepatic expression of CCAAT/enhancer binding protein α: hormonal and metabolic regulation in rats. Diabetologia. 1997; 40(10):1117–24. 9349591 10.1007/s001250050796

[52] Croniger CM, Millward C, Yang J, Kawai Y, Arinze IJ, Liu S. Mice with a deletion in the gene for CCAAT/enhancer-binding protein beta have an attenuated response to cAMP and impaired carbohydrate metabolism. J Biol Chem. 2001; 276(1):629–38. doi: 10.1074/jbc.M007576200 11024029 10.1074/jbc.M007576200

[53] Hayhurst G, Lee Y, Lambert G, Ward JM, Gonzalez F. Hepatocyte Nuclear Factor 4α (Nuclear Receptor 2A1) is essential for maintenance of hepatic gene expression and lipid homeostasis. Mol Cell Biol. 2001; 21(4):1393–403. doi: 10.1128/MCB.21.4.1393-1403.2001 11158324 10.1128/MCB.21.4.1393-1403.2001 PMC99591

[54] Gupta RK, Kaestner KH. HNF-4alpha: from MODY to late-onset type 2 diabetes. Trends Mol Med. 2004; 10(11):521–4. doi: 10.1016/j.molmed.2004.09.004 15519277 10.1016/j.molmed.2004.09.004

[55] Yamagata K, Furuta H, Oda N, Kaisaki PJ, Menzel S, Cox NJ, et al Mutations in the hepatocyte nuclear factor-4alpha gene in maturity onset diabetes of the young (MODY1). Nature. 1996; 384(6608):458–60. doi: 10.1038/384458a0 8945471 10.1038/384458a0

[56] Gautier-Stein A, Zitoun C, Mithieux G, Rajas F. A distal region involving hepatocyte nuclear factor4α and CAAT/enhancer binding protein markedly potentiates the protein kinase A stimulation of the glucose-6-phosphatase promoter. J Mol Endocrinol. 2005; 19(1):163–74. 10.1210/me.2004-0105 15388792

[57] Hanson RW. Regulation of phosphoenolpyruvate carboxykinase (GTP) gene expression. Annu Rev Biochem. 1997; 66:581–611. doi: 10.1146/annurev.biochem.66.1.581 9242918 10.1146/annurev.biochem.66.1.581

[58] Sever R, Glass CK. Signaling by nuclear receptors. Cold Spring Harb Perspect Biol. 2013; 5(3):a016709 doi: 10.1101/cshperspect.a016709 23457262 10.1101/cshperspect.a016709 PMC3578364

[59] Fang Y, Liu HX, Zhang N, Guo GL, Wan YJ, Fang J. NURBS: a database of experimental and predicted nuclear receptor binding sites of mouse. Bioinformatics. 2013; 29(2):295–7. doi: 10.1093/bioinformatics/bts693 23196988 10.1093/bioinformatics/bts693 PMC3546791

[60] Jitrapakdee S, Slawik M, Medina-Gomez G, Campbell M, Wallace JC, Sethi JK. The peroxisome proliferator-activated receptor-gamma regulates murine pyruvate carboxylase gene expression in vivo and in vitro. J Biol Chem. 2005; 280(29):27466–76. doi: 10.1074/jbc.M503836200 15917242 10.1074/jbc.M503836200 PMC4304003

[61] Yoon JC, Puigserver P, Chen G, Donovan J, Wu Z, Rhee J, et al Control of hepatic gluconeogenesis through the transcriptional coactivator PGC-1. Nature. 2001; 413 (6852):131–8. doi: 10.1038/35093050 11557972 10.1038/35093050

[62] Pei L, Waki H, Vaitheesvaran B, Wilpitz DC, Kurland IJ, Tontonoz P. NR4A orphan nuclear receptors are transcriptional regulators of hepatic glucose metabolism. Nat Med. 2006; 12(9):1048–55. doi: 10.1038/nm1471 16906154 10.1038/nm1471

[63] Rhee J, Inoue Y, Yoon JC, Puigserver P, Fan M, Gonzalea F, et al Regulation of hepatic fasting response by PPARγ coactivator-1α (PGC-1α): Requirement for hepatocyte nuclear factor 4α in gluconeogenesis. Proc Natl Acad Sci USA. 2003; 100(7):4012–7. doi: 10.1073/pnas.0730870100 12651943 10.1073/pnas.0730870100 PMC153039

[64] Holloway MG, Miles GD, Dombkowski AA, Waxman DJ. Liver-specific hepatocyte nuclear factor-4α deficiency: greater impact on gene expression in male than in female mouse liver. Mol Endocrinol. 2008; 22(5):1274–86. doi: 10.1210/me.2007-0564 18276827 10.1210/me.2007-0564 PMC2366185

